# Structure modification of luteolin and the influence of its derivatives on biological activities

**DOI:** 10.3389/fnut.2025.1546932

**Published:** 2025-03-12

**Authors:** Lingyang Kong, Wei Wu, Chenliang Li, Lengleng Ma, Junbai Ma, Meitong Pan, Shan Jiang, Weili Liu, Jiao Xu, Wei Ma

**Affiliations:** ^1^College of Pharmacy, Heilongjiang University of Chinese Medicine, Harbin, China; ^2^College of Jiamusi, Heilongjiang University of Chinese Medicine (TCM), Jiamusi, China

**Keywords:** hemp seeds, luteolin derivatives, structural modification, psoriasis, anti-inflammation, antioxidation

## Abstract

**Introduction:**

This research aims to synthesize luteolin derivatives from hemp seeds by means of chemical synthesis, improve the synthesis process, simplify the procedure, and increase the yield to obtain new luteolin derivatives. Additionally, anti-inflammatory and antioxidant activities of hemp seed extracts and newly synthesized substances are tested to screen out substances with high anti-inflammatory and antioxidant activities.

**Methods:**

Using luteolin as the raw material, acetyl, propionyl, and butyryl groups are introduced into the molecular structure of luteolin. A one-pot synthesis method is employed to modify the hydroxyl groups at positions 5, 7, 3′, and 4′ to obtain six new luteolin acyl derivatives. The molar ratio of reaction conditions is 1:4. Pyridine (20 mL) is used as the solvent, and the reaction is carried out at 25°C and 110°C. Exploring the anti-inflammatory and antioxidant activities of luteolin and its derivatives by establishing a psoriasis model.

**Results:**

The products are separated and purified by column chromatography and recrystallization, and six new luteolin acyl derivatives were synthesized: namely, 7,3′,4′-tri-O-acetylated luteolin (A), 7,3′,4′-tri-O-propionylated luteolin (B), 7,3′,4′-tri-O-butyrylated luteolin (C), 5,7,3′,4′-tetra-O-acetylated luteolin (D), 5,7,3′,4′-tetra-O-propionylated luteolin (E), and 5,7,3′,4′-tetra-O-butyrylated luteolin (F). By establishing a psoriasis like mouse model, the results showed that luteolin and its derivatives have good therapeutic effects on inflammation and antioxidation.

**Discussion:**

Six new acyl derivatives of luteolin were synthesized through structural modification, which improved their solubility and bioavailability. In the psoriasismodel, it has been proven that acyl derivatives of luteolin have anti-inflammatory and antioxidant activities, and have a relieving effect on psoriasis.Provide theoretical basis and potential treatment strategies for the future treatment of psoriasis.

## 1 Introduction

Hemp seeds are the dried and mature fruits of *Cannabis sativa* L. of the Moraceae family and are traditional crops with both medicinal and edible properties. Hemp seeds are sweet and neutral in nature, and enter the spleen, stomach, and large intestine meridians. They have the effect of moistening the intestines and promoting defecation and are used for blood deficiency and fluid depletion, and intestinal dryness and constipation. According to literature reports, hemp seeds contain various unsaturated fatty acids. In addition, there are also lignanamides (1), cannabinoids (2), flavonoids, steroids and terpenes, alkaloids and other components. Modern pharmacological studies have shown that hemp seeds have effects such as antioxidation, anti-aging, anti-fatigue, anti-inflammation, neuroprotection, lipid regulation and liver protection, and immune regulation (3).

Luteolin is a typical natural flavonoid compound (4). It is widely distributed in nature and mainly exists in traditional Chinese medicinal materials such as honeysuckle, chrysanthemum, schizonepeta, herba ajugae, artichoke, callicarpa nudiflora, and hemp seeds. Luteolin also exists in various vegetables and fruits.

The C ring of luteolin is the key active component for antioxidation and scavenging free radicals, especially C′3-OH. The B-cyclic catechol structure of luteolin can contribute hydrogen electrons (H+), stabilize free radicals, thereby clearing harmful free radicals, protecting human cells or tissues, and delaying human aging. To further enhance the biological activity of luteolin, scholars have carried out various structural adjustments and modifications. In recent years, the research focus has mainly been on structural modifications such as alkylation, acylation, and salt formation on its phenolic hydroxyl groups (5).

**Figure 1:**
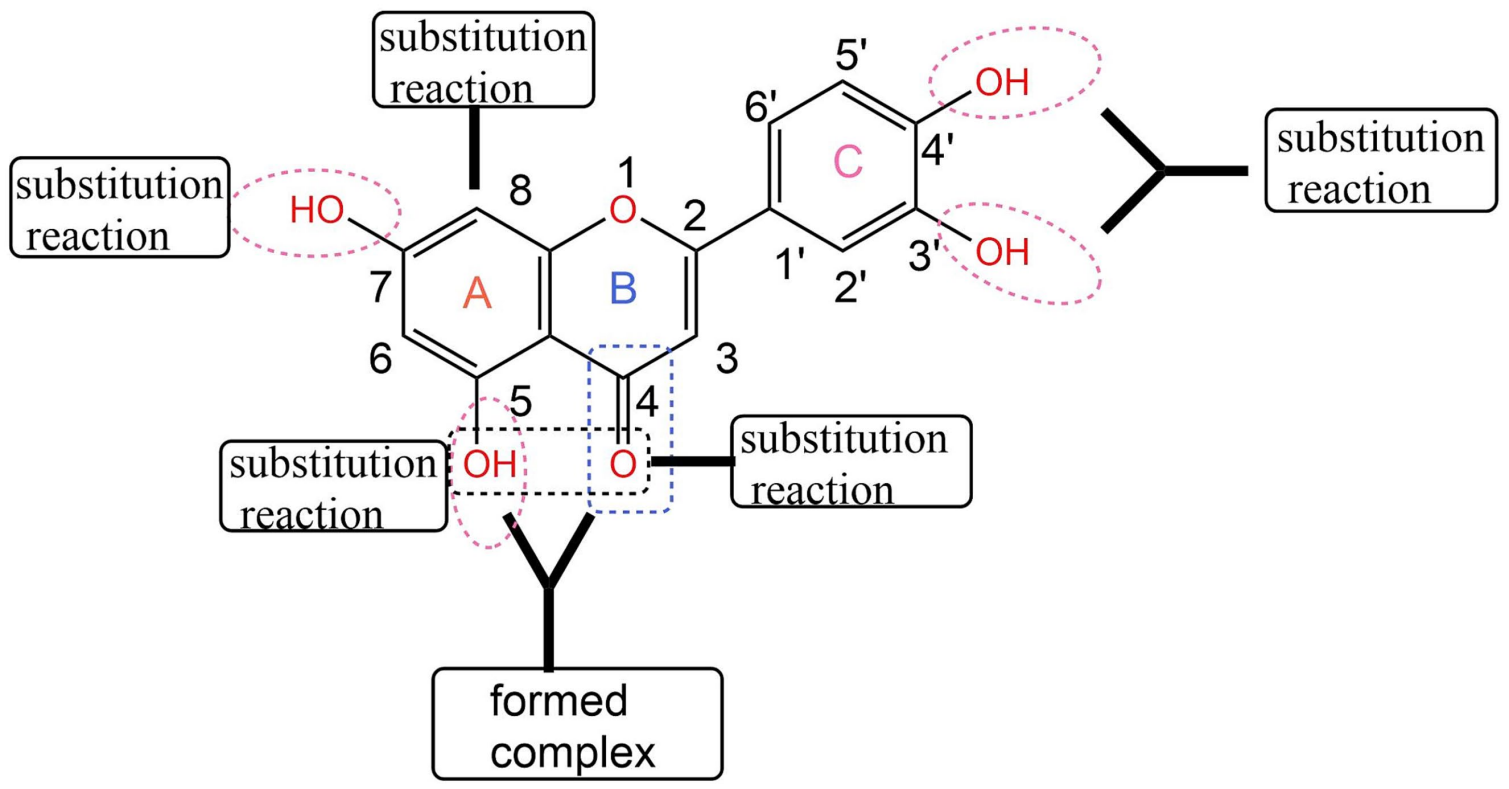
Structural modification of luteolin (43).

Due to its excellent antioxidant (6), anti-inflammatory (7), and anticancer properties, luteolin has become the focus of natural drug research and application at home and abroad in recent years. In order to further improve the biological activity of luteolin, its structure has been modified. The most studied are substitution reactions such as alkylation and acylation at its active phenolic hydroxyl sites (8). The basic skeleton of luteolin is formed by two benzene rings A and B connected through a central three-carbon atom. The C7 on ring A and C′3 and C′4 on ring B all have active phenolic hydroxyl groups, but the phenolic hydroxyl group on C′3 is slightly less active than that on C7 and C′4. Wang Qiqin (9) used a one-pot method to remove active hydrogen, making this structure easy to undergo a nucleophilic reaction with RBr, and then obtain a mono-substituted compound of luteolin. Lü Pengcheng (10) first used 1,2-dibromoethane to form ring protection for C′3-OH and C′4-OH on its B ring, and then carried out various modification treatments on C7-OH on the A ring, such as alkylation and amination. Zhang Jiange (11) first protected C′3-OH and C′4-OH with dichlorodiphenylmethane, then introduced an alkane chain at the C7 position. Through the action of an aqueous solution of glacial acetic acid or Pd (OH)2/C and H2, the protecting group was successfully removed and the selective retention of active hydroxyl groups was achieved. He Yaoyao (12) and others first carried out selective benzylation of luteolin with benzyl chloride, then carried out alkylation or acylation reactions, and finally obtained a series of 5-O-substituted derivatives by catalytic hydrogenolysis. Luteolin can undergo substitution reactions at different positions, as shown in Figure 1.

Using luteolin as the parent compound, structural modifications are carried out on the hydroxyl groups at positions 5, 7, 3′, and 4′ to obtain a series of new acylated derivatives, which improve its low solubility and bioavailability. The luteolin acylated derivatives obtained through chemical structure modification can broaden its biological activity and application range, providing new ideas for its research in anti-inflammatory, antioxidant, and other fields, providing experimental data for the development and application of new anti-inflammatory drugs, and also providing a theoretical basis for the derivatization of other flavonoid compounds.

Psoriasis, known as “Baibi” in traditional Chinese medicine, has typical symptoms including erythema accompanied by scales and itching (13). Psoriasis is a systemic inflammatory disease. Its pathogenesis is caused by the release of immune-related cells (such as macrophages, neutrophils, and T cells), the increase in pro-inflammatory cytokines (such as TNF-*α*, IL-17A, IL-23), and the chronic activation of the innate and adaptive immune systems, which leads to long-term damage to multiple tissues (such as skin tissue) and organs (such as the spleen) (14). It is mainly affected by two factors, namely genes and environment, including radiation, drugs, and microbial infections, etc., but there are differences in different regions (15).

Research has found that luteolin can inhibit various acute and chronic inflammations (16). Hall (17) also obtained similar findings when studying the structure–activity relationship of the anti-inflammatory activities of sesquiterpenes. When the exocyclic methylene group of Helenalin was saturated to form 2,3-Dihydrohelenalin, the inhibitory effect on rat paw swelling induced by carrageenan decreased from 75 to 38%.

Therefore, we took luteolin as the parent compound and introduced acetic anhydride, propionic anhydride, and butyric anhydride groups into the molecular structure of luteolin. We adopted the one-pot method for synthesis. Through the optimization of the synthesis process, the most suitable reaction conditions were screened out. Finally, the products were separated and purified by column chromatography and recrystallization methods. In this way, animal experiments were carried out to verify whether the six new luteolin derivatives still possess the anti-inflammatory and antioxidant activities of luteolin.

## 2 Experimental materials and methods

### 2.1 Grouping, modeling and administration methods

The animals used in the experiment were female BALB/c mice at the SPF level (7–8 weeks old, weighing 20–25 g). The experimental mice were raised in a pathogen-free animal room and had free access to food and water. The experimental mice were randomly divided into 17 groups, with 8 mice in each group. The backs of the mice were depilated using an electric shaver and depilatory cream, and relevant experiments were carried out 24 h later. In the experiment, on the backs of the mice in the model group (IMQ group), tacrolimus group (TK group), high-dose luteolin (Lut-H) group, high-dose luteolin derivative groups A-H, B-H, C-H, D-H, E-H, F-H, low-dose luteolin (Lut-L) group and low-dose luteolin derivative groups A-L, B-L, C-L, D-L, E-L, F-L, 62.5 mg of 5% imiquimod cream was applied every day. Four hours after the model was established, the positive drug tacrolimus (TK group) cream was evenly applied to the backs of the mice. The administration method of this positive drug was carried out concerning the existing literature and papers (12). For the administration groups, the luteolin solution at a dose of 80 mg/kg/d (Lut-H) and the luteolin derivative solutions at a dose of 80 mg/kg/d for the high-dose luteolin derivative groups were applied on the backs. For the low-dose luteolin (Lut-L) group and its low-dose luteolin derivative groups, the luteolin solution and derivative solutions at a dose of 40 mg/kg/d were, respectively, applied on the backs. Then, the blank group and the model group were smeared with petrolatum as a control. The mice were sacrificed on the 7th day of the experiment. Luteolin and its derivatives were formulated using polyethylene glycol 400. Skin samples from the backs of the mice were taken and thoroughly cleaned with a physiological saline solution. Part of them was fixed in 4% paraformaldehyde solution, and the other part was stored in a freezer at −80°Cto prepare for the subsequent experiments.

### 2.2 PASI score

After the application of the medication, the degree of psoriasis vulgaris symptoms on the back of the mice was scored by taking photos the next day (18). The PASI score is based on three indicators: scales, erythema, and infiltration, and is evaluated according to the severity of the symptoms. The degree of silver-like symptoms on the back skin of mice was ultimately determined based on the scoring results of scales, erythema, and infiltration (19).

### 2.3 H&E staining

Soak the back skin of mice in 4% paraformaldehyde, then dehydrate the tissue with different concentrations of alcohol for transparency treatment. Subsequently, wax immersion treatment is carried out. The tissue was immersed in a mixture of xylene and paraffin, followed by three replacements with paraffin for 1 h each time. Put the skin tissue into the embedding machine for embedding. The embedded wax block is placed on a slicer for slicing, with a slice thickness of 4 *μ* m. Before dewaxing, dry the slices in an oven for 30 min, then deparaffinize them with xylene and hydrate them in different concentrations of alcohol. After the above steps, the slices were placed in a staining solution (hematoxylin Harris) and stained for 5 min. After staining, the slices were sliced with xylene and finally sealed for observation (20).

### 2.4 Spleen index

The spleen is not only an important lymphoid organ but also the main place where T cells and B cells survive. Moreover, it is a crucial area for capturing antigens, recognizing antigens, and triggering immune responses. This organ can also indirectly reflect the degree of psoriasis-like symptoms. At the end of the last day of the experiment, the mice were treated with the cervical dislocation technique and were dissected thoroughly using biological dissection tools such as scissors, dissecting forceps and tweezers. During this process, we took out the spleens of the mice, photographed them and weighed to calculate the spleen index (21).

### 2.5 Enzyme-linked immunosorbent assay (ELISA) determination

Immunoassay technology relies on the highly selective and specific recognition and binding mechanism generated by specific antibodies with antigens or haptens, and is mainly used for accurate analysis and determination of the antibodies or antigens to be tested. Approximately 0.15 g of skin tissue from the backs of mice in each group stored in a −80°C freezer was taken out. Firstly, the tissue on the backs of the mice needed to be cut into small pieces and then placed into 1.5 mL EP tubes. After that, 1.3 mL of cell lysis buffer was added and vortexed to mix evenly. Next, an ice bath was carried out for 30 min to fully lyse these cells. Finally, centrifuge for 15 min to collect the supernatant sample. ELISA kits containing IL-6 (serial number H007-1-2, antigen sequence number Uniprot NO: p08505) and TNF - *α* (serial number H052-1-2, antigen sequence number Uniprot NO: p06804) were used to detect proteins in mouse back skin tissue samples (all kits were purchased from Jiancheng, Nanjing, China).

### 2.6 Determination of SOD enzyme activity and measurement of GSH and MDA contents in skin tissue

An appropriate amount of skin tissue from the backs of mice was taken from the −80°C freezer and thawed on ice. A 100 mg portion of the tissue was then placed into a pre-cooled phosphate buffer solution and ground evenly using a tissue grinder. Then add 9 times the volume of normal saline for dilution to prepare a 10% skin tissue homogenate. Set the high-speed centrifuge to centrifuge at 3000 rpm for 10 min at 4°C. Collect the supernatant after each centrifugation and repeat this process twice. The samples are then ready for the experiment. SOD (22), GSH (23), MDA (24) antioxidant assay kits were used to measure mouse skin tissue. The determination of various indicators was carried out according to the instructions of the kit [SOD (A001-3-2), GSH (A006-2-1) and MDA (A003-1-2) all kits were purchased from Jiancheng, Nanjing, China].

### 2.7 Data processing

Data were subjected to statistical analysis using the mean ± standard deviation. The significant differences between data were analyzed using the Graphpad Prism (25) software. *p*-value of less than 0.05 was considered statistically significant.

## 3 Result

### 3.1 Impact on the degree of skin lesions in psoriasis mouse models

**Figure 2:**
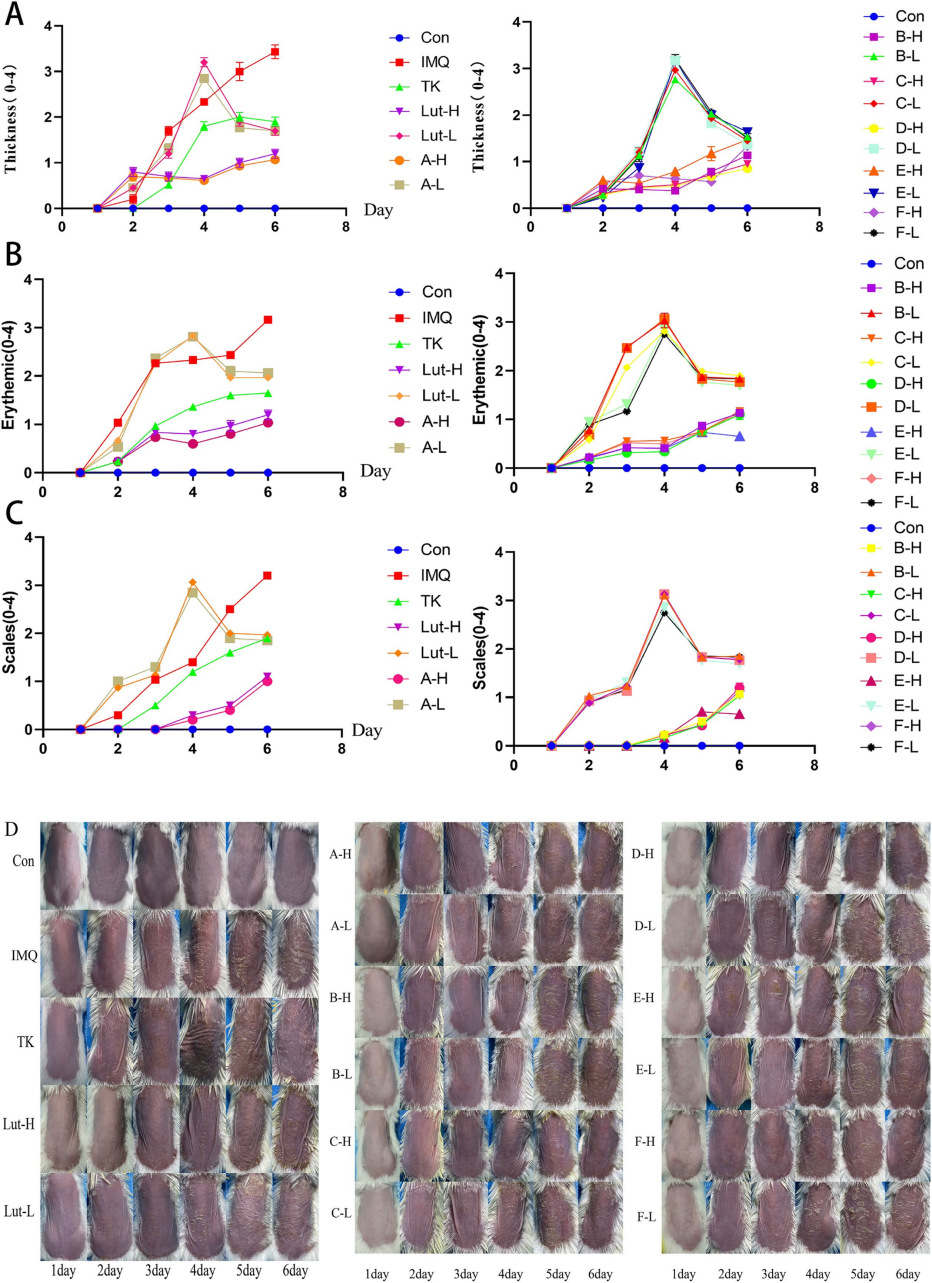
**(A)**, **(B)** and **(C)**: The PASI score for the back skin of mice, **(D)**: Changes in the back skin of a mice psoriasis injury model induced by imiquimod.

As shown in Figure 2, through the comparative analysis of the back skin conditions of mice in different groups, the healthy skin condition of the mice in the normal group can be clearly seen. The color of the backs of the mice gradually changed from the original light pink to a deeper and more extensive dark red, as if it were a sign of some kind of inflammatory reaction deep within the skin.

It is worth noting that on the 6th day after the use of the imiquimod drug, this change reached its peak. For the mice in the high-dose luteolin group and the high-dose luteolin derivative groups A, B, C, D, E, and F, the situation was slightly different. As shown in Figure 2D, there was smooth skin, a reduction in the thickening of the stratum corneum (Figure 2A), superficial erythema (Figure 2B), and a decrease in scales (Figure 2C). The low-dose luteolin group and the low-dose derivative groups A, B, C, D, E, and F did not seem to effectively alleviate the symptoms related to psoriasis. The severity of skin damage was evaluated based on the statistical data of the PASI score (Figures 2A–D). The scores of the normal group smeared with petrolatum were all 0. In the mice treated with imiquimod, the scores regarding psoriasis, cortex thickness and the degree of erythema all showed a gradually increasing pattern. As the number of days of drug administration increased, the PASI scores of the mice in the high-dose luteolin (80 mg/kg/d) group and the high-dose luteolin derivative groups A, B, C, D, E, and F in terms of erythema, scales and cortex thickness gradually decreased over time, which was similar to the performance of the mice in the tacrolimus group. Compared with the high-dose luteolin group, the high-dose luteolin derivative groups B, C, and D had similar effects and even surpassed the high-dose luteolin group.

### 3.2 Impact on the skin pathological changes of the psoriasis mouse model

**Figure 3:**
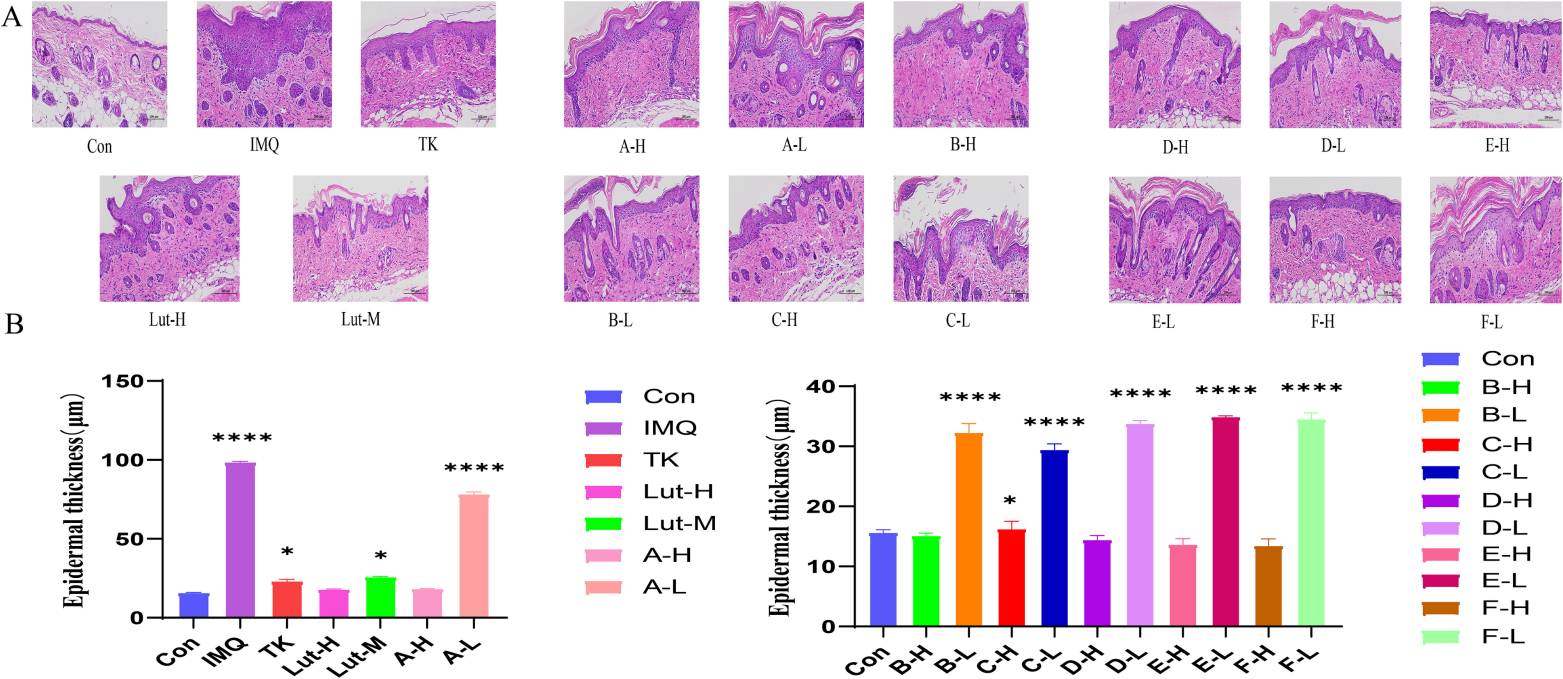
**(A)**: H&E staining pattern of imiquimod induced back psoriasis in mice, **(B)**: Skin epidermal thickness on the back of mice. (****) *p* < 0.0001 and (*) *p* < 0.05 versus control group.

Through histopathological analysis, the back skin samples of mice in each group were subjected to H&E staining (Figures 3A,B). In Figure 3, it was observed that the keratinocytes in the blank group appeared in an orderly arrangement, indicating that they maintained a normal morphological and functional state in the tissue structure. This pathological feature reflects the balance and homeostasis of the mouse skin under the physiological environment. However, compared with the mice in the blank group, significant changes occurred on the backs of the mice with the psoriasis model induced by the application of imiquimod (26). The spinous cell layer of the epidermis not only thickened but was also accompanied by the downward extension of the epidermal layer and further thickening of the stratum corneum.

The results of H&E staining revealed that both high-dose luteolin and its derivatives exhibited significant anti-inflammatory effects. These compounds not only successfully inhibited the thickening of the stratum corneum and Munro’s microabscess lesions caused by imiquimod but also effectively reduced the infiltration of inflammatory cells. Compared with the high-dose luteolin group, the high-dose groups of B-H and C-H performed even better in treating skin damage. However, when compared with the model group, the low-dose luteolin group and the luteolin derivative groups could also inhibit the epidermal thickening in mice induced by imiquimod. Although the phenomenon of Munro’s microabscess occurred, which slightly damaged the structure of the epidermal layers, this thin barrier system still demonstrated its powerful resistance. In addition, this epidermal layer was also striving to reduce the keratinization process of the epidermis and tried its best to maintain the softness and smoothness of the skin surface. It indicates that the low-dose luteolin group and the luteolin derivative groups can also inhibit inflammatory cells.

### 3.3 Impact on the immune organs of the psoriasis mouse model

**Figure 4:**
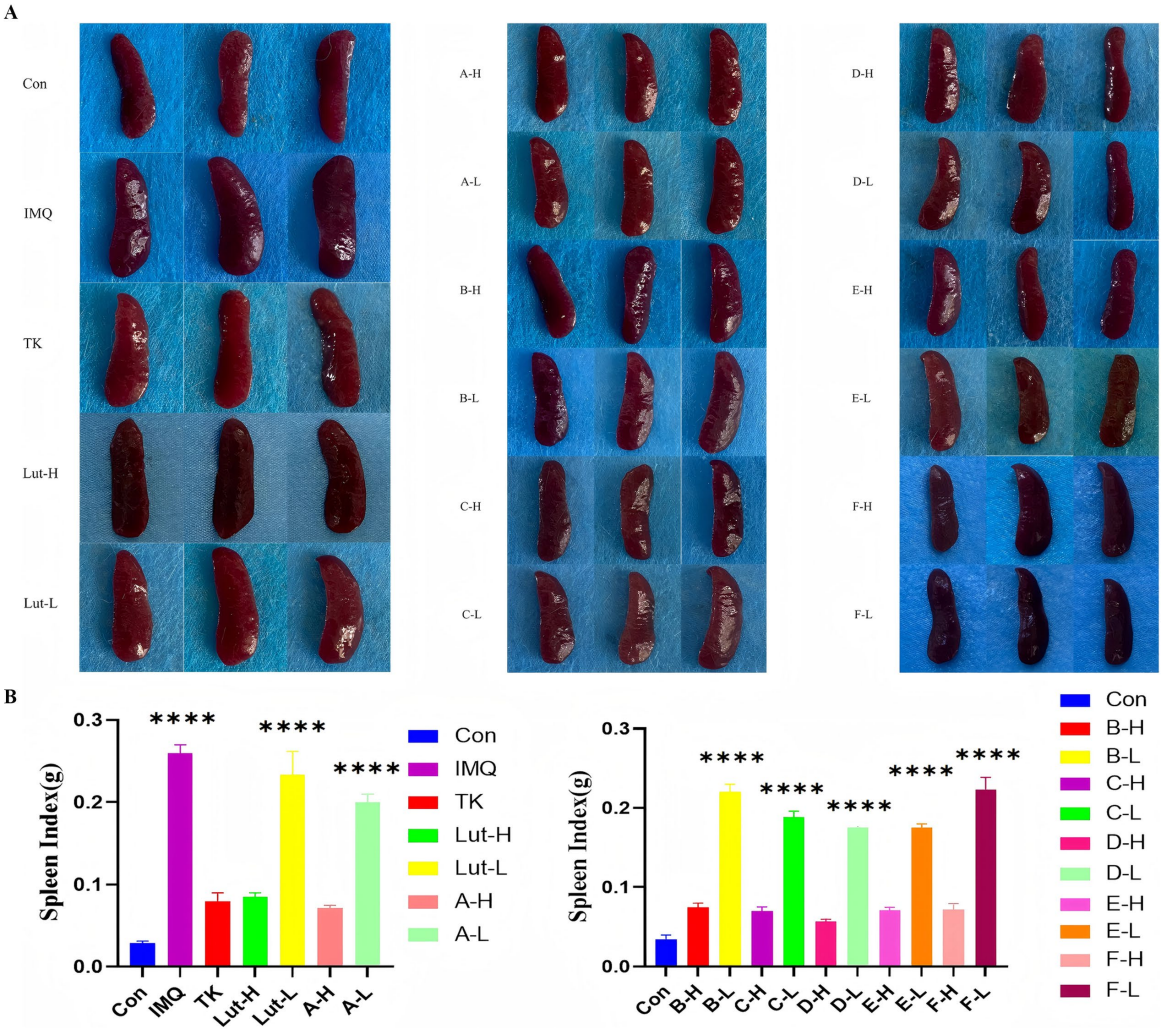
**(A)**: Appearance map of spleens in each group of mice, **(B)**: spleen index map of mice in each group. (****) *p* < 0.0001 versus control group.

The results shown in Figure 4A indicate that, compared with the mice in the blank group, the volume of the spleens of the mice in the model group increased significantly. However, it is worth noting that although the spleen index of the model group was significantly higher than that of the blank group, when we delved deeper into the high-dose luteolin group and the high-dose luteolin derivative groups, we found that the spleens of these mice did not show the same volume increase phenomenon. On the contrary, their spleen indices (the ratio of spleen weight to body weight) were comparable to or even lower than that of the earlier blank group. The results revealed that the high-dose groups of C-H and D-H derivatives showed significant effects in alleviating the skin inflammation induced by imiquimod (Figure 4B).

### 3.4 Impact on skin inflammatory factors of the psoriasis mouse model

**Figure 5:**
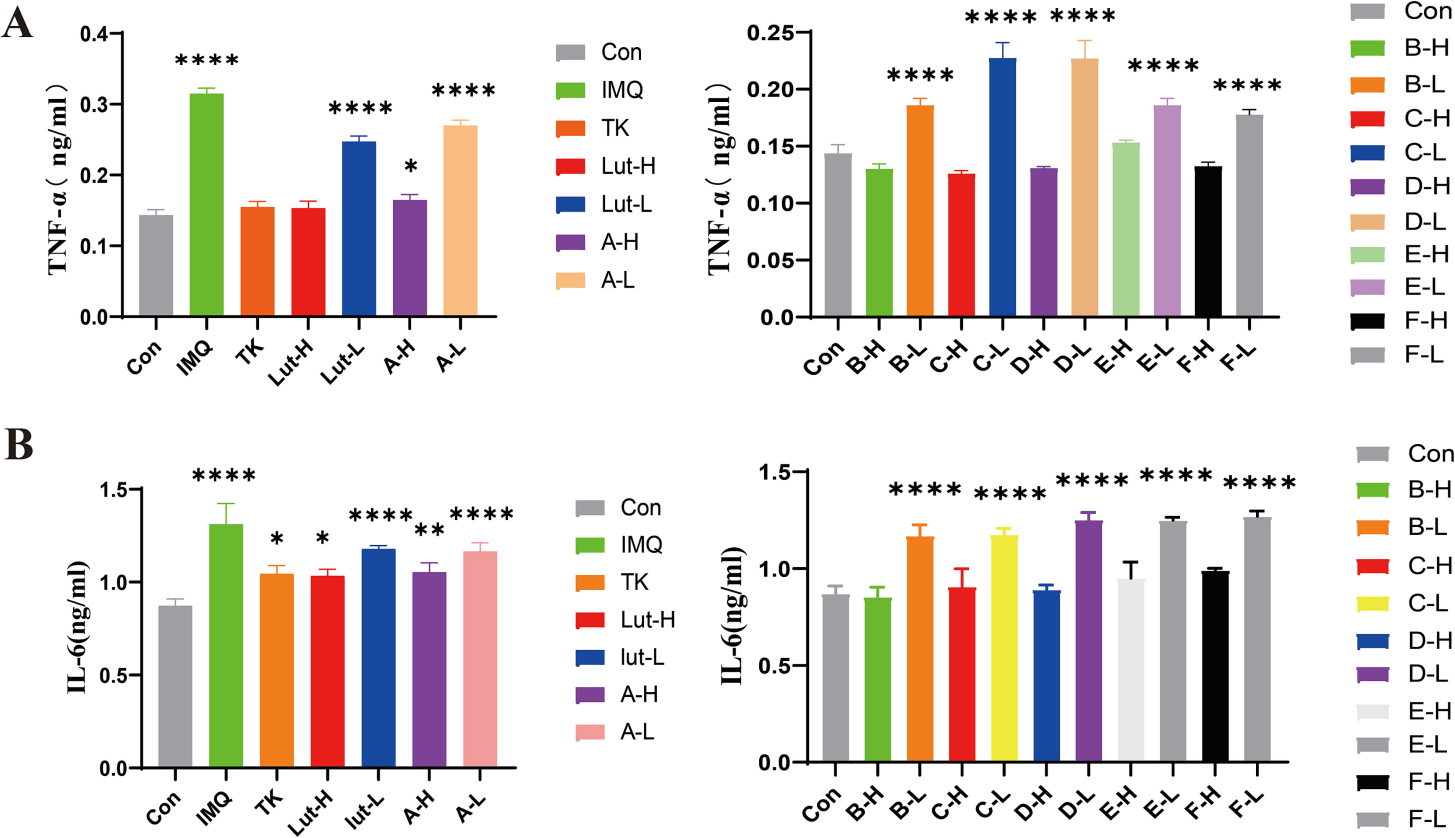
**(A)**: TNF-*α* at the back skin of mice Protein expression content, **(B)**: IL-6 at the back skin of mice Protein expression content. (****) *p* < 0.0001 and (*) *p* < 0.05 versus control group.

The enzyme-linked immunosorbent assay (ELISA) (27) was used to determine the changes in the contents of inflammatory factors in the skin lesions of psoriasis-like mice, to confirm that luteolin and its related derivatives have inhibitory effects on these inflammatory cytokines. The results shown in Figure 5A indicate that, compared with the control group, the expressions of the inflammatory cytokines TNF-*α* and IL-6 in the model group were significantly increased. However, the expressions of factors such as TNF-α and IL-6 in the positive drug group were significantly lower than those in the model group. The inhibitory effects of luteolin and its derivatives A-H, B-H, C-H, and F-H groups (80 mg/kg/d) on IL-6 were roughly the same as those of tacrolimus. In particular, the D-H and E-H groups had better effects in inhibiting inflammatory factors than the positive drug group. This reveals the potential therapeutic effects of luteolin and its derivatives on psoriasis, a common chronic skin disease (Figure 5B).

### 3.5 Impact on SOD activity, MDA and GSH contents in the supernatant of mouse skin tissue

**Figure 6:**
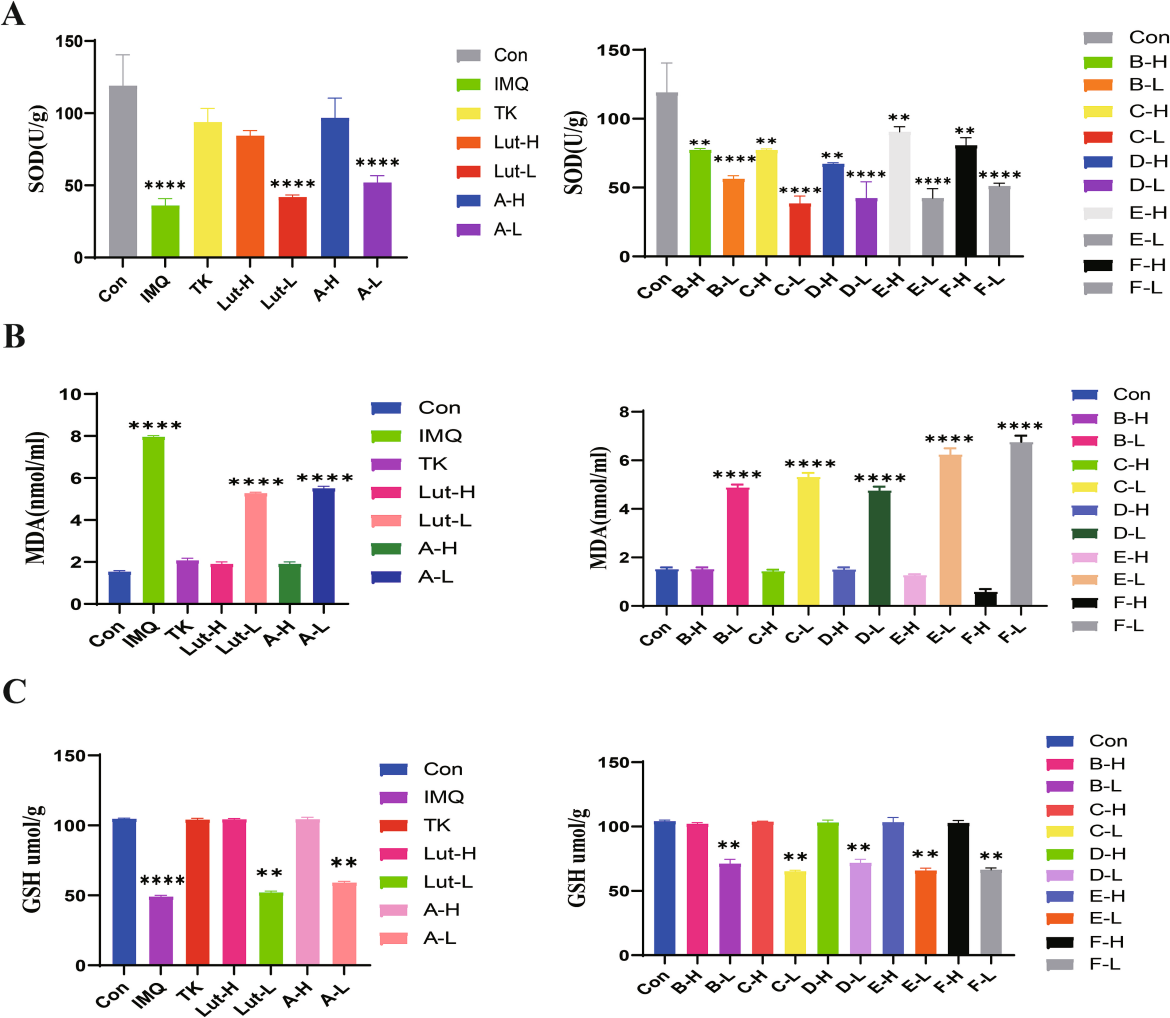
**(A)**: The effects of SOD enzyme activity in mouse back skin, **(B)**: The effects of MDA content in mouse back skin, and **(C)**: the effects of GSH content in mouse back skin.

The free-radical theory states that in the human body’s antioxidant defense system, GSH and SOD antioxidant enzymes, as well as small - molecule non - enzymatic antioxidants and the generation of free radicals in the body are in a dynamic equilibrium (28). When this balance is disrupted, peroxides are produced in the body. Therefore, we measured the antioxidant indices. As observed in Figure 6A, when taking the blank group as a reference, the contents of SOD and GSH in the skin tissue of the mice in the model group decreased, while the content of MDA increased. Compared with the model group, the contents of SOD and GSH increased (Figures 6B,C), which led to a gradual improvement in the scavenging ability of GSH, and the content of lipid peroxidation products such as MDA decreased. The antioxidant activities of luteolin and its derivatives were better than those of the positive drug tacrolimus. In particular, the luteolin derivative groups A-H and E-H had better effects on increasing the content of SOD than the positive drug group. The luteolin derivative groups C-H and F-H had good effects on reducing the content of MDA, which were almost the same as those of the blank group mice. The luteolin derivative groups B-H and D-H had good effects on increasing the content of GSH, which were almost the same as the effects of the positive drug tacrolimus. Therefore, luteolin and its derivatives have a good antioxidant effect on the skin.

## 4 Experiment

### 4.1 Synthetic route of trisubstituted-O-acylation reaction

**Figure 7:**
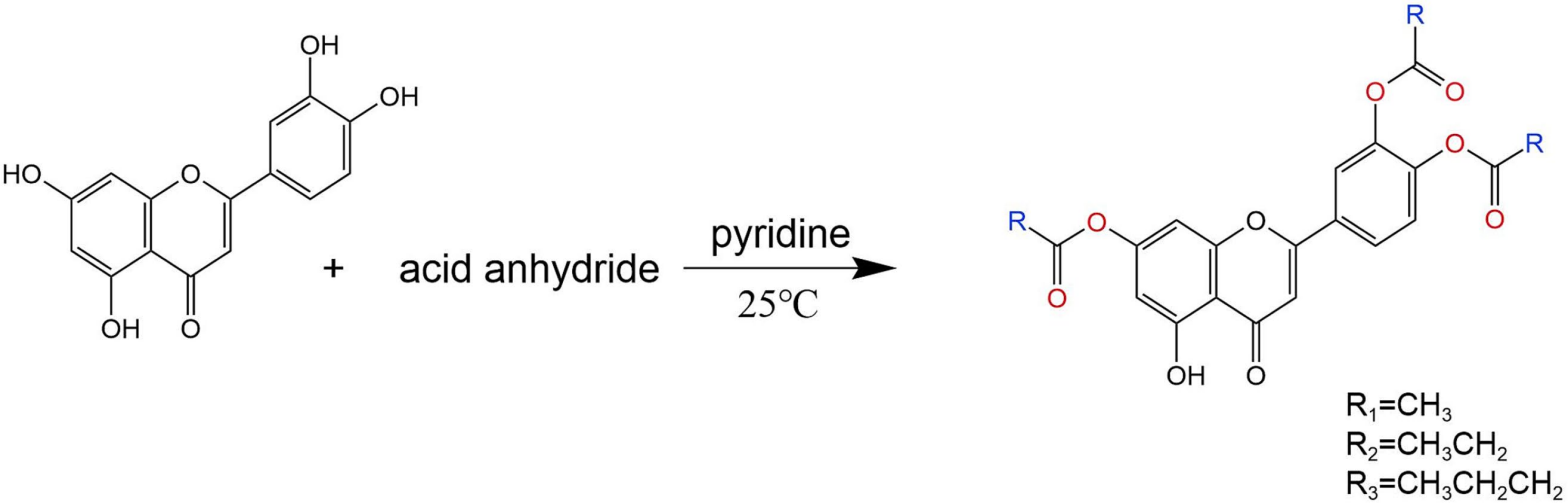
Synthesis roadmap of three substituted derivatives of luteolin.

The structure of luteolin was modified with luteolin as the parent compound. The synthesis of trisubstituted derivatives A-C of luteolin with acetic anhydride, propionic anhydride and butyric anhydride is shown in the synthetic pathway in Figure 7.

### 4.2 Synthetic route of tetrasubstituted-O-acylation reaction

**Figure 8:**
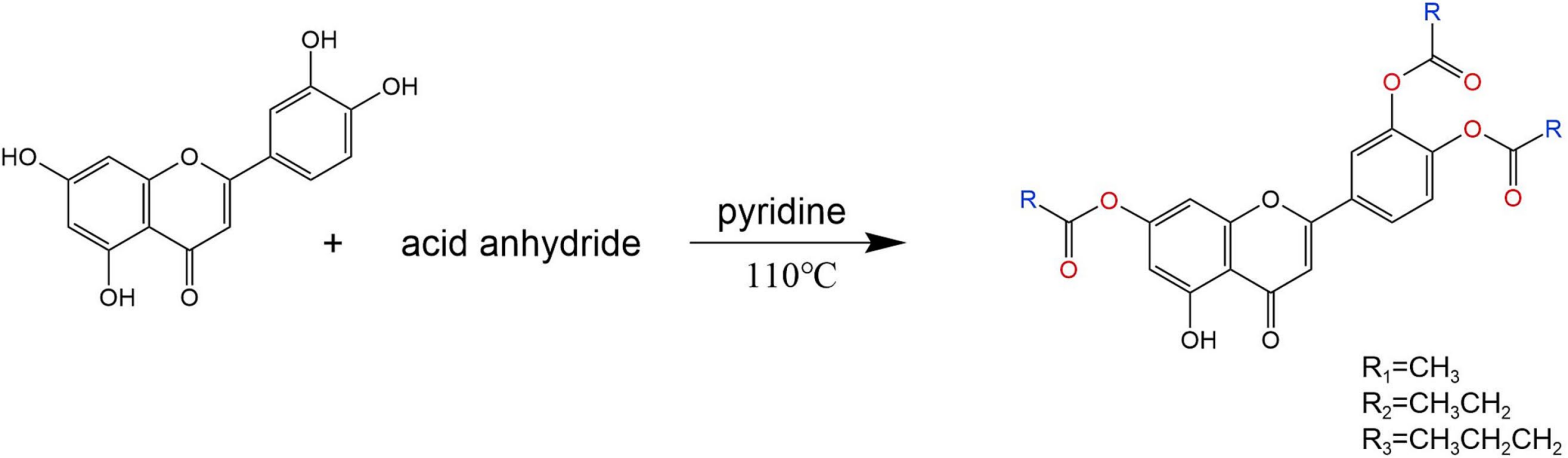
Synthesis roadmap of four substituted derivatives of luteolin.

The synthesis of tetrasubstituted derivatives D-F of luteolin with acetic anhydride, propionic anhydride and butyric anhydride is shown in the synthetic pathway in Figure 8.

### 4.3 Structural characterization methods of luteolin derivatives

^1^H NMR spectra were recorded on Bruker 400 MHz spectrometer using DMSO-d6 as solvent. Mass spectra were recorded on an ESI mass spectrometer. IR spectra were recorded with Bruker Tensor 27 series FT-IR spectrophotometer in KBr disks.

### 4.4 Target compound synthesis method

#### 4.4.1 7,3′,4′-tri-O-acetylated luteolin (A)

Weigh 1 g (3.5 mmol) of luteolin and place it in a triangular flask. Add 20 mL of pyridine, stir with an electromagnetic stirrer, and react at 25°C. After the reaction solution becomes clear, add an appropriate amount of acetic anhydride to continue the reaction. Stir at room temperature for 1 h, and detect the reaction by TLC. The developing agent is ethyl acetate: methanol = 1:1–4:1 (v/v). Filter the reaction mixture, separate the filtrate using a silica gel column, and elute with a gradient of ethyl acetate/methanol. Combine the eluents containing the product to recover the eluent, and recrystallize the residue from methanol to obtain a yellow solid product. The drying temperature is 180°C, the yield is 72%. m.p = 180°C–180.1°C; IR(KBr)v: 3510, 3,078, 2,935, 1735, 1,489, 1,188, ^1^HNMR(400 MHz, DMSO-d6)δ12.90–12.66(m, 1H), 8.09–8.07 (m, 1H), 8.06–8.00 (m, 1H), 7.527.49 (m, 1H), 7.16 (s, 1H), 7.11 (t, J = 2.0 Hz, 1H), 6.67(t, J = 2.0 Hz, 1H), 2.34–2.30 (m, 9H, 3 × CH_3_).

#### 4.4.2 7,3′,4′-tri-O-propionylated luteolin (B)

Weigh 1 g (3.5 mmol) of luteolin and place it in a triangular flask. Add 20 mL of pyridine, stir with an electromagnetic stirrer, and react at 25°C. After the reaction solution becomes clear, add an appropriate amount of propionic anhydride to continue the reaction. Stir at room temperature for 1 h, and detect the reaction by TLC. The developing agent is ethyl acetate: methanol = 1:1–4:1 (v/v). Filter the reaction mixture, separate the filtrate using a silica gel column, and elute with a gradient of ethyl acetate/methanol. Combine the eluents containing the product to recover the eluent, and recrystallize the residue from methanol to obtain a yellow solid product. The drying temperature is 140°C, the yield is 76%. m.*p* = 144°C-150°C; IR(KBr)v: 3387, 3,082, 2,980, 1785, 1,492, 1,188, ^1^H NMR(400 MHz, DMSO-d6) *δ* 12.90–12.66(m, 1H), 8.06–8.03 (m, 1H), 8.03–8.00(m, 1H), 7.50–7.49 (m, 1H), 7.16 (s, 1H), 7.10 (t, J = 2.0 Hz, 1H), 6.65 (t, J = 2.0 Hz, 1H), 2.26(m, 6H, 3 × CH_2_), 1.12 (m, 9H, 3 × CH_3_).

#### 4.4.3 7,3′,4′-tri-O-butyrylated luteolin (C)

Weigh 1 g (3.5 mmol) of luteolin and place it in a triangular flask. Add 20 mL of pyridine, stir with an electromagnetic stirrer, and react at 25°C. After the reaction solution becomes clear, add an appropriate amount of succinic anhydride to continue the reaction. Stir at room temperature for 1 h, and detect the reaction by TLC. The developing agent is ethyl acetate: methanol = 1:1–4:1 (v/v). Filter the reaction mixture, separate the filtrate using a silica gel column, and elute with a gradient of ethyl acetate/methanol. Combine the eluents containing the product to recover the eluent, and recrystallize the residue from methanol to obtain a yellow solid product. The drying temperature is 150°C, the yield is 75%. m.*p* = 157°C-162°C； IR(KBr)v: 3510, 3,082, 2,966, 1782, 1,492, 1,125,^1^H NMR(400 MHz, DMSO-d6)*δ* 12.83–12.61(m,1H), 8.13–7.92(m, 2H), 7.52–7.43(m, 1H), 7.18(s, 1H), 7.11 (t, J = 1.9 Hz, 1H), 6.65 (t, J = 1.9 Hz, 1H), 2.67–2.55 (m, 6H, 3 × CH2), 1.70–1.50 (m, 6H, 3 × CH_2_), 1.08–0.85 (m, 9H, 3 × CH_3_).

#### 4.4.4 5,7,3′,4′-tetra-O-acetylated luteolin (D)

Weigh 1 g (3.5 mmol) of luteolin and place it in a triangular flask. Add 20 mL of pyridine and stir with an electromagnetic stirrer for 10 min. Allow the reaction to proceed at room temperature. Once the reaction solution is clear, add an appropriate amount of acetic anhydride and continue the reaction at 110°C until the reaction stops. The reaction was detected by TLC, and the developing agent was ethyl acetate: methanol = 1:1–4:1 (v/v). Filter the reaction mixture, separate the filtrate using a silica gel column, and elute with a gradient of ethyl acetate/methanol. Combine the eluents containing the product to ecover the eluent, and recrystallize the residue from methanol to obtain a yellow solid product. The drying temperature is 220°C, the yield is 78%. m.*p* = 226°C-227°C； IR(KBr)v: 3510, 3,070, 2,939, 1774,1,508,1,188,^1^H NMR (400 MHz, DMSO-d6) *δ* 8.06–7.96 (m, 2H), 7.61 (d, J = 4.5 Hz,1H), 7.48 (dd, J = 8.0, 4.0 Hz, 1H), 7.12–7.03 (m, 1H), 6.93 (s,1H), 2.35–2.30 (m,12H,4 × CH_3_).

#### 4.4.5 5,7,3′,4′-tetra-O-propionylated Luteolin (E)

Weigh 1 g (3.5 mmol) of luteolin and place it in a triangular flask. Add 20 mL of pyridine and stir with an electromagnetic stirrer for 10 min. Allow the reaction to proceed at room temperature. Once the reaction solution is clear, add an appropriate amount of succinic anhydride and continue the reaction at 110°C until the reaction stops. The reaction was detected by TLC, and the developing agent was ethyl acetate: methanol = 1:1–4:1 (v/v). Filter the reaction mixture, separate the filtrate using a silica gel column, and elute with a gradient of ethyl acetate/methanol. Combine the eluents containing the product to recover the eluent, and recrystallize the residue from methanol to obtain a yellow solid product. The drying temperature is 160°C, the yield is 82%. m.*p* = 165°C-166°C；IR(KBr)v: 3522, 3,082, 2,985, 1785, 1,504, 1,125, ^1^H NMR (400 MHz, DMSO-d6) *δ* 8.10–7.97 (m, 2H), 7.63 (t, J = 1.9 Hz,1H), 7.54–7.40 (m, 1H), 7.07 (t, J = 1.9 Hz,1H), 6.94 (s, 1H), 2.72–2.57 (m, 8H, 4 × CH_2_), 1.21–1.02 (m, 12H, 4 × CH_3_).

#### 4.4.6 5,7,3′,4′-tetra-O-butyrylated luteolin (F)

Weigh 1 g (3.5 mmol) of luteolin and place it in a triangular flask. Add 20 mL of pyridine and stir with an electromagnetic stirrer for 10 min. Allow the reaction to proceed at room temperature. Once the reaction solution is clear, add an appropriate amount of succinic anhydride and continue the reaction at 110°C until the reaction stops. The reaction was detected by TLC, and the developing agent was ethyl acetate: methanol = 1:1–4:1 (v/v). Filter the reaction mixture, separate the filtrate using a silica gel column, and elute with a gradient of ethyl acetate/methanol. Combine the eluents containing the product to recover the eluent, and recrystallize the residue from methanol to obtain a yellow solid product. The drying temperature is 150°C, the yield is 83%. m.*p* = 150°C-151°C；IR(KBr)v: 3487, 3,090, 2,970, 1779, 1,508, 1,188,^1^H NMR (400 MHz, DMSO-d6) *δ* 8.06–7.95 (m, 2H), 7.62 (d,J = 1.8 Hz, 1H), 7.55–7.39 (m, 1H), 7.05 (t,J = 1.8 Hz, 1H), 6.95 (s, 1H),2.68–2.55 (m,8H,4 × CH_2_), 1.79–1.56(m,8H,4 × CH_2_),1.03–0.87 (m,12H,4 × CH_3_). The above spectra are all shown in Supplementary Figure 1.

## 5 Discussion

To obtain new luteolin derivatives, this study mainly took luteolin as the parent compound and used acetic anhydride, propionic anhydride and butyric anhydride as raw materials for synthesis by the one-pot method. This synthesis procedure is simple, the reaction is controllable, the products are easy to separate, and there are few by-products. Through the optimization of reaction conditions, the yield of the acylated products of luteolin has been improved. Moreover, the solubility of these products in aqueous solutions and alcohol solutions was investigated. The solubility of the acylated products A, B, C, D, E, and F in hot water is slightly better than that of luteolin, with product A having the best solubility. Compared with the solubility in aqueous solutions, the acylated products A, B, C, and D have better solubility in polyethylene glycol solutions. And their structures were characterized by means of infrared spectroscopy, hydrogen nuclear magnetic resonance spectroscopy and other technical means. The results showed that the synthesis of this kind of compound laid a good foundation for further chemical modifications on the 5th, 7th, 3′, and 4′ positions of luteolin compounds.

In our study, luteolin and a series of luteolin derivatives have a significant inhibitory effect on imiquimod-induced psoriasis in mice. Though the Psoriasis Area and Severity Index (PASI) score, spleen index and hematoxylin and eosin (H&E) staining, it was demonstrated that the acylated derivatives of luteolin can improve skin damage, reduce the infiltration of inflammatory factors, and have a significant anti-inflammatory effect. The acylated derivatives of luteolin inhibit imiquimod-induced psoriasis lesions in mice by down-regulating the expressions of TNF-*α* and IL-6 in the mouse skin tissue, increasing the contents of superoxide dismutase (SOD) and glutathione (GSH), and reducing the content of malondialdehyde (MDA). Among them, the high-dose groups of luteolin derivatives such as A-H, E-H and F-H exert the same antioxidant effect as tacrolimus, and even the groups of derivatives B-H, C-H and D-H are superior to the effects of the positive drug tacrolimus and the high-dose group of luteolin. They can not only significantly alleviate the skin symptoms of mice with psoriasis but also show a good repairing effect on various tissue damages caused by psoriasis. This finding provides brand-new ideas and directions for the research and development of drugs related to psoriasis.

There are many synthetic methods for luteolin derivatives, mainly including modifications of hydroxyl groups, carbonyl groups, and modifications of A and B rings. Through the optimization of synthetic methods, luteolin derivatives with good solubility and high bioavailability can be obtained. Lo (29) designed nine acyl derivatives with different structures and obtained tri-O-benzyl luteolin through chemical reactions between themselves and benzyl bromide. On this basis, by adding acyl chloride - triethylamine as raw materials, derivatives of 5-O-acyl tri-O-benzyl luteolin were synthesized. Fischer (30) reacted luteolin with an excess of fatty acid acyl chlorides and obtained tetra-acylated luteolin derivatives. Zhou Meirong (31) carried out the Mannich reaction between the H bond at the C-8 position of ring A of luteolin and primary aliphatic amines in an aqueous solution of acetaldehyde and discovered ten new 8-aminomethylated derivatives.

In this study, the one-pot method was used to conduct structural modifications on the hydroxyl groups at the 5th, 7th, 3′ and 4′ positions of luteolin, and a total of six new acylated derivatives of luteolin were synthesized. This synthesis method is simple to operate and has a high yield. In the experiment, the influences of reaction temperature and reaction time on the synthesis were investigated. It was found that if the reaction temperature was too low, the reaction could not proceed. On the other hand, if the temperature was too high, the by-products of the reaction would increase, resulting in a low yield. Moreover, different luteolin derivatives were synthesized at different temperatures. The acylated products A, B, and C were synthesized at 25°C, while the acylated products D, E, and F were synthesized at 110°C. The reaction time had little impact on this experiment. Based on conditions such as the clarity of the solution during the reaction, the yield of derivatives, and the reduction of substitution by-products, 90 min was selected as the optimal reaction time. To compare the one-pot method with the synthesis methods recorded in the literature, there was no difference in yield. However, the method in literature (32) was complicated, required strict control of conditions, and was difficult in separate. In contrast, the one-pot method was relatively simple in design, easy to control the conditions, and easy to separate. The water solubility and lipid solubility of luteolin were poor. Luteolin was almost insoluble in cold water and slightly soluble in hot water. Osonga (33) reacted luteolin with dibenzyl diphosphate and synthesized luteolin tetraphosphate. The solubility of this compound in water is 297 times that of luteolin. Tsai (34) solved the problem of the difficulty in dissolving luteolin in water and synthesized three water-soluble luteolin phosphate substances, laying a foundation for the development of its derivatives. Among the six luteolin derivatives generated in this chapter, after testing, it was found that the water solubilities of derivatives A and B were better than that of luteolin, and the solubilities of derivatives A, B, C, D, E and F in alcohol solutions were all better than that of luteolin, indicating that the introduction of acetic anhydride and propionic anhydride groups increased the solubility of luteolin.

Currently, in the treatment of psoriasis, the chemical components found in traditional Chinese medicine have been isolated and used as independent extracts or monomers. In this chapter, we applied imiquimod on the backs of mice to create a psoriasis-like model, aiming to study the inhibitory effect of luteolin and its derivatives on psoriasis-like symptoms. From different perspectives, we preliminarily explored the role of luteolin and luteolin derivatives in inhibiting psoriasis-like symptoms using ELISA experiments and other methods.

Previous studies have found that luteolin can significantly inhibit cell growth, remarkably reduce skin oxidative stress, and exhibit excellent antioxidant properties (35, 36). Based on the results analysis of the Psoriasis Area and Severity Index (PASI) score, spleen measurement and hematoxylin and eosin (H&E) staining methods in this study, it was discovered that both high-dose and low-dose luteolin and luteolin derivatives can alleviate scales, erythema and the thickness of the cortex, relieve the symptoms of splenomegaly, and reduce the occurrence of microabscess lesions in the model group. This implies that luteolin and luteolin derivatives A, B, C, D, E, and F have a certain inhibitory effect on psoriasis-like symptoms. This is consistent with previous studies (37). Based on the above research findings, luteolin has shown a repairing effect on the skin damage caused by psoriasis, which means that it has the potential to become a topical drug for the treatment of psoriasis.

Studies have shown that luteolin exhibits excellent anti-inflammatory effects and can inhibit the release of various inflammatory factors, thereby alleviating the inflammatory response of tissues (38, 39). In the psoriasis animal model, there are a large number of inflammatory cells, such as IL-6, TNF-*α*, etc. The ELISA method was used to detect the expression of inflammatory factors in the skin lesion tissues of mice. In addition, the oxidative stress response accelerates the inflammatory response through a variety of signal pathways, including NF-κB and mitogen-activated protein kinases. Studies have found that antioxidants can regulate psoriasis symptoms, further indicating that oxidative stress plays an important role in psoriasis (40). It protects cell membranes from damage by scavenging hydroxyl radicals, hydrogen peroxide, and peroxides. MDA is used as an indicator to evaluate the level of lipid peroxidation. In various pathological and skin damage model groups of psoriasis, its level exceeds that of the blank group. Therefore, MDA is regarded as a key indicator for evaluating the condition of psoriasis and the curative effect of drug treatment (41). GSH has the function of scavenging free radicals and can slow down the lipid peroxidation reaction (42). This study found that luteolin and luteolin derivatives can down-regulate the expressions of inflammatory factors such as IL-6 and TNF-*α*, can also increase the activity of SOD enzyme and the content of GSH in the skin tissue induced by imiquimod, and at the same time can reduce the content of MDA, further inhibit the release of free radicals, thereby enhancing the body’s anti-inflammatory and antioxidant abilities and having the effect of treating psoriasis.

## 6 Conclusion

The structure of luteolin has been carefully adjusted and modified, and six acyl derivatives of luteolin have been successfully synthesized. By analyzing the influences of reaction time, reaction temperature, and the molar amounts of luteolin, acetic anhydride, propionic anhydride, and butyric anhydride on the synthesis of acyl derivatives, the optimal synthesis conditions were finally determined. Moreover, through the structural analysis of the acyl derivatives of luteolin, it can be known that such derivatives have formed stable structures and possess good solubility. Therefore, it has also been confirmed in the psoriasis model that the acyl derivatives of luteolin possess anti-inflammatory and antioxidant activities and exhibit the effect of alleviating psoriasis. It has been found that compounds A-H, E-H, and F-H have a good effect on alleviating psoriasis, which is equivalent to the efficacy of luteolin and tacrolimus, while the activities of B-H, C-H, and D-H are higher than that of the positive drug tacrolimus. The results of spectral analysis (IR, ^1^H NMR) have verified all the synthesized compounds.

## Data Availability

The datasets presented in this study can be found in online repositories. The names of the repository/repositories and accession number(s) can be found in the article/Supplementary material.
