# Supplementary material for: Structure modification of luteolin and the influence of its derivatives on biological activities
Source: Front Nutr. 2025 Mar 12;12:1546932. doi: 10.3389/fnut.2025.1546932 (PMC11936824; doi:10.3389/fnut.2025.1546932)

7,3 ', 4' - tri-O-acetyl luteolin（A）IR


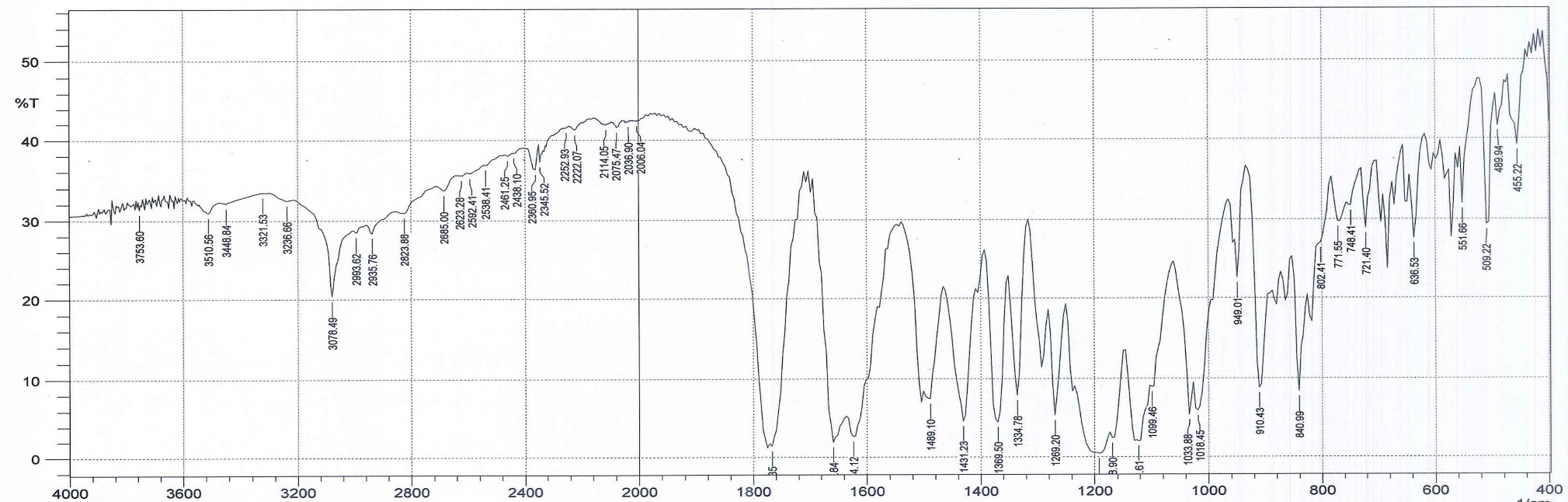


7,3 ', 4' - tri-O-propylated luteolin（B）IR


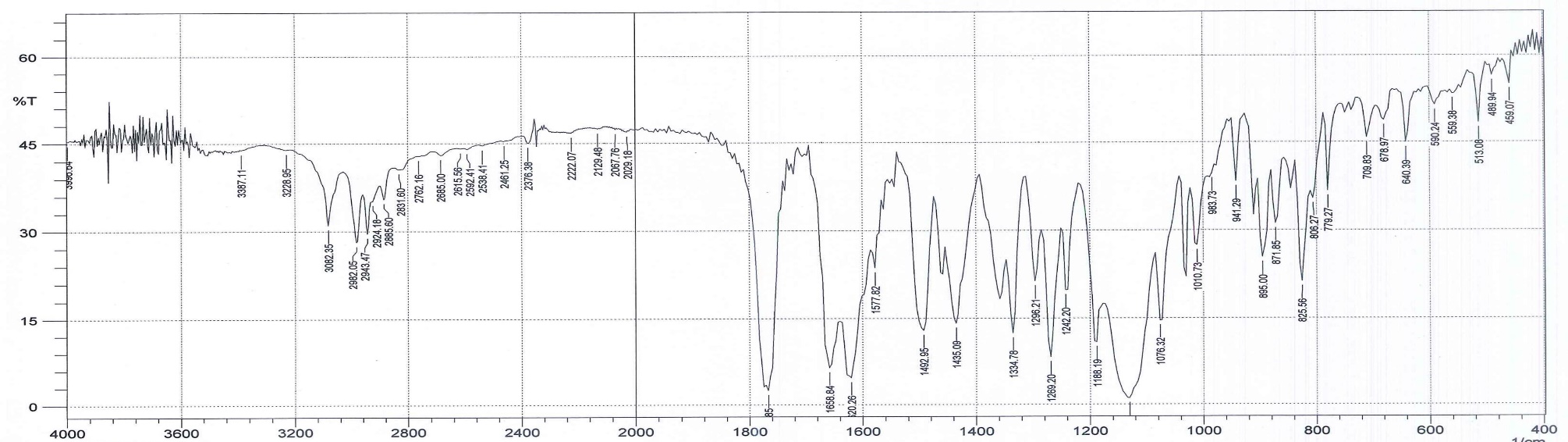


7,3 ', 4' - tri-O-acetyl luteolin（C）IR


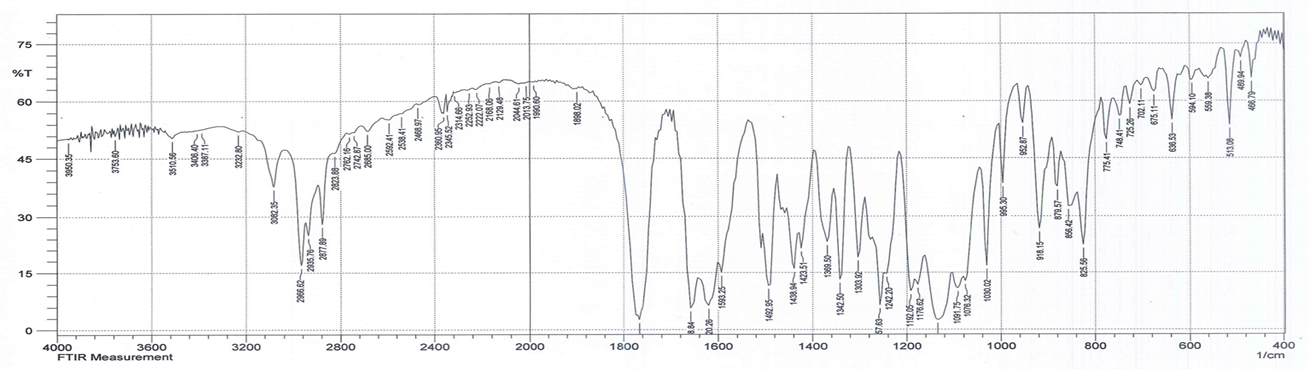


5,7,3 ', 4' - tetra-O-acetyl luteolin（D）IR


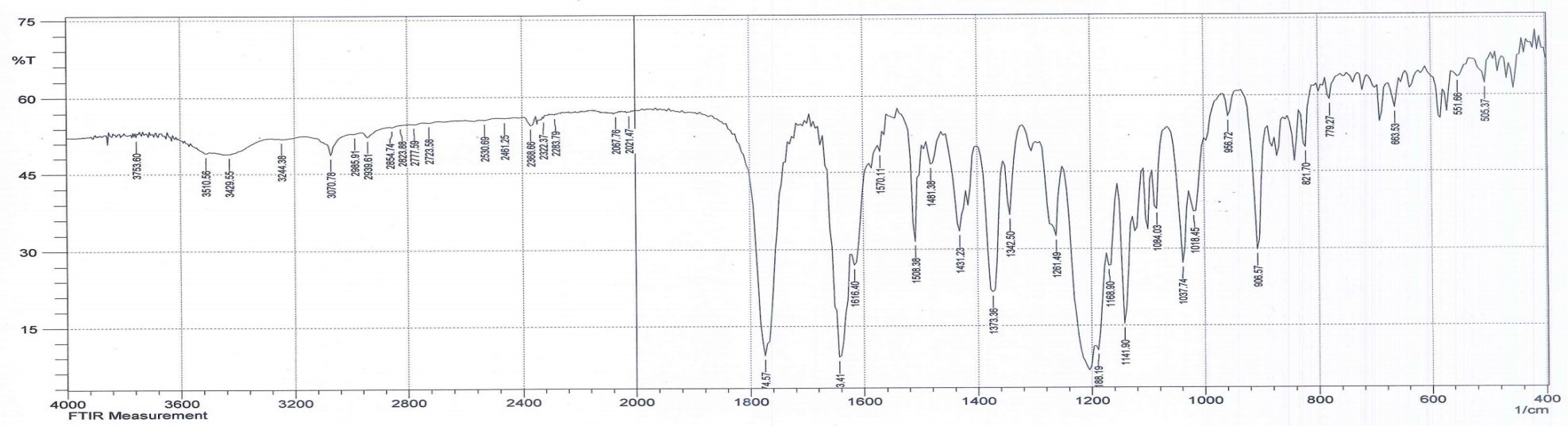


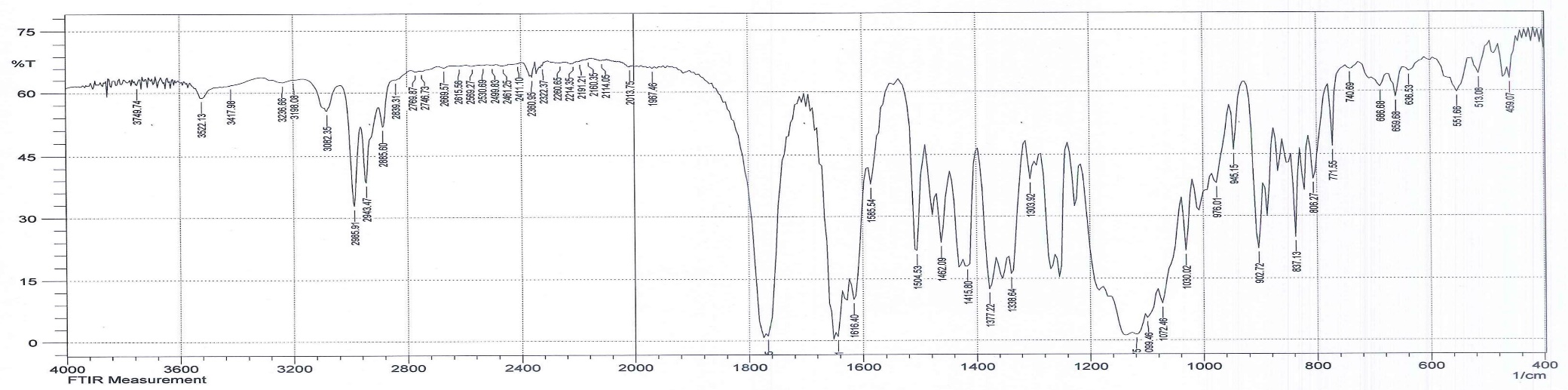
5,7,3 ', 4' - tetra-O-propionylated luteolin（E）IR

5,7,3 ', 4' - tetra-O-butyrylated luteolin（F）IR


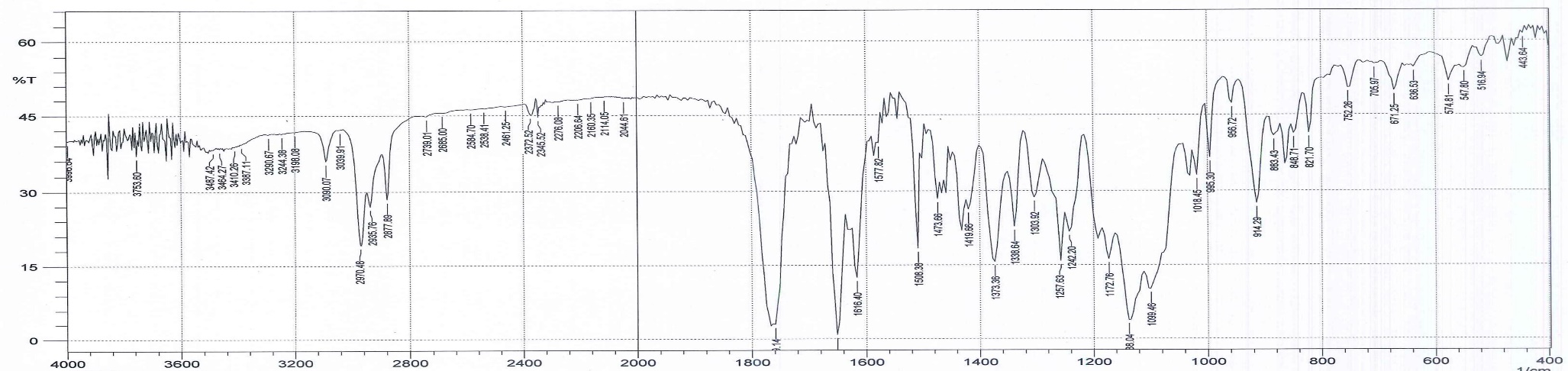


7,3 ', 4' - tri-O-acetyl luteolin（A）^1^HNMR


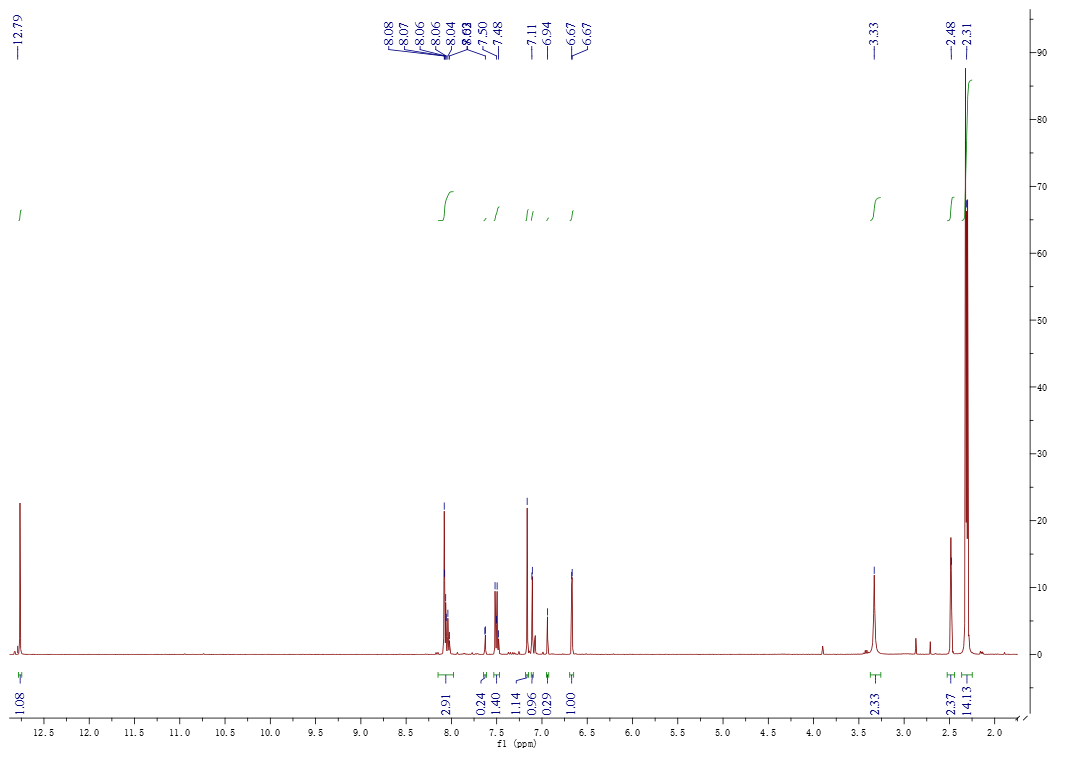


7,3 ', 4' - tri-O-propylated luteolin（B）^1^HNMR


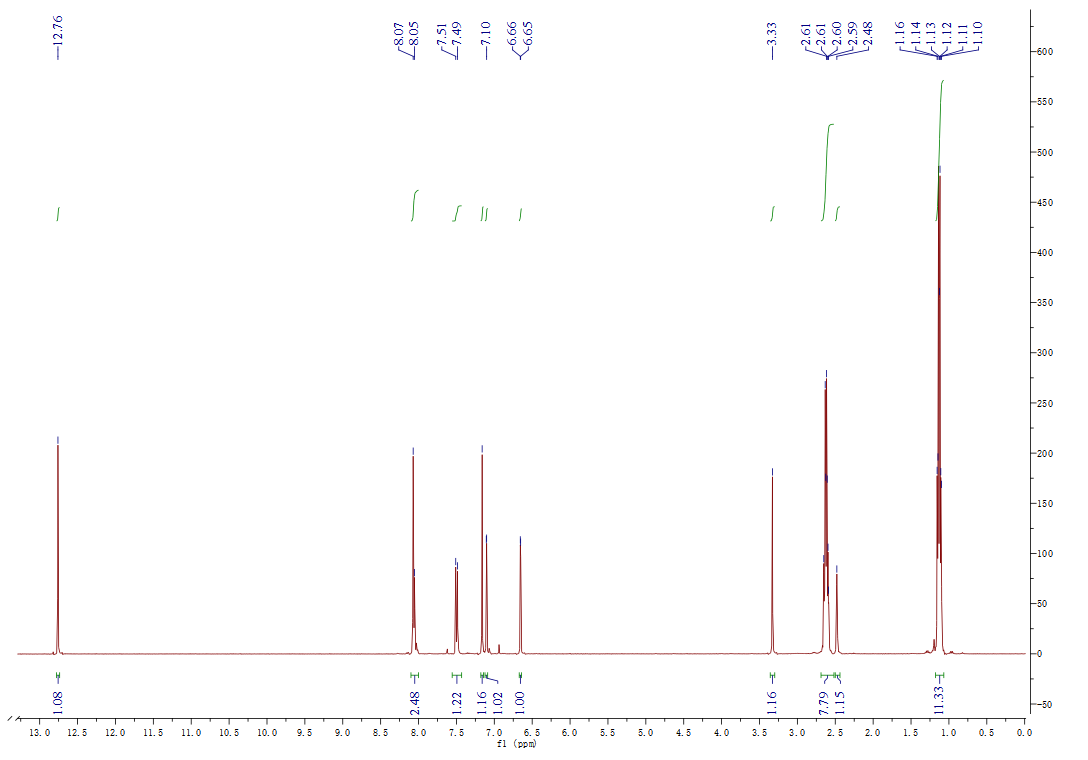


7,3 ', 4' - tri-O-butyrylated luteolin（C）^1^HNMR


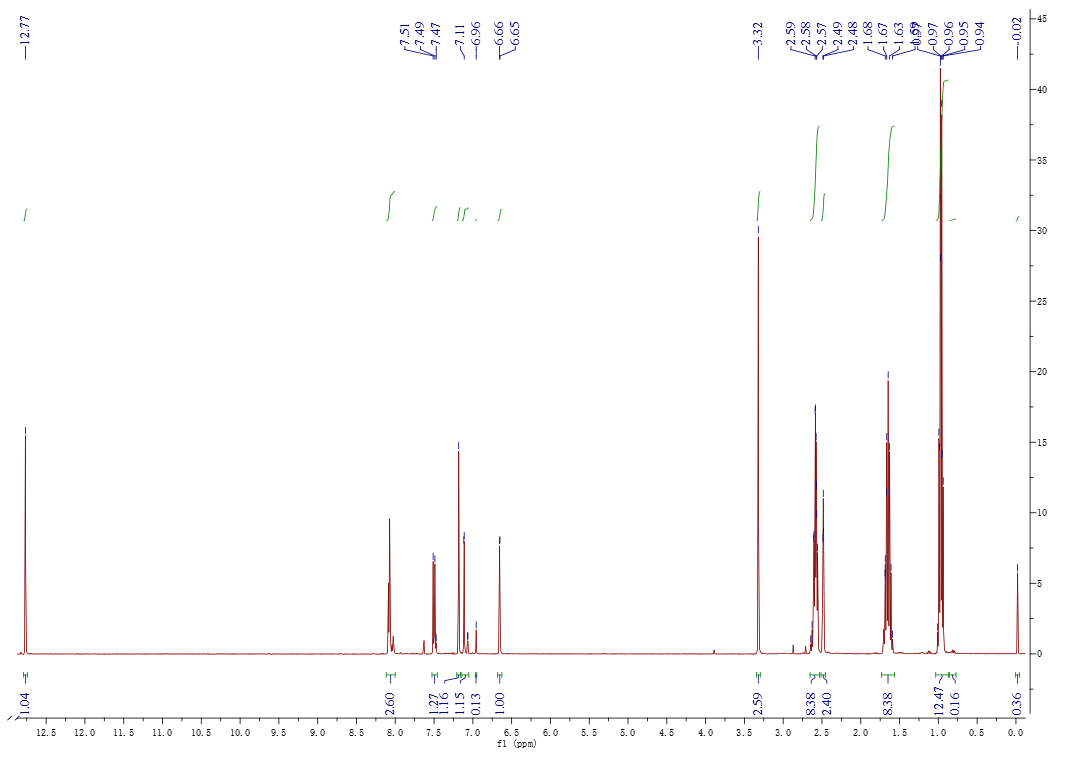


5,7,3 ', 4' - tetra-O-acetyl luteolin（D）^1^HNMR


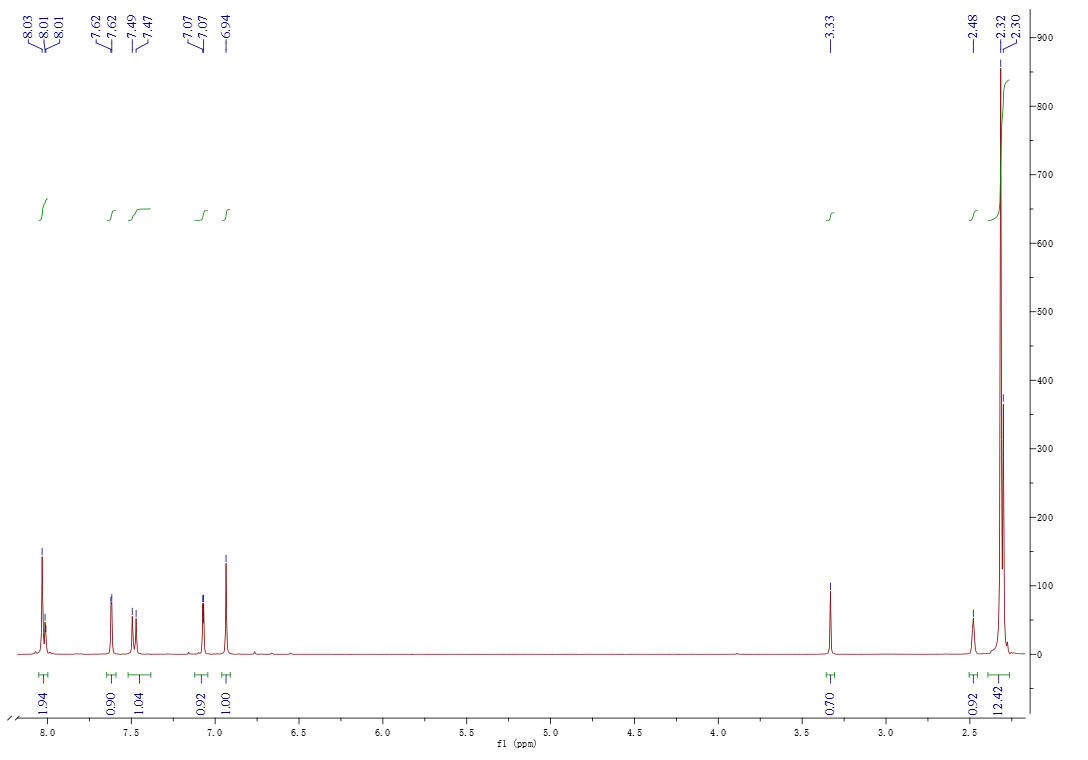


5,7,3 ', 4' - tetra-O-propionylated luteolin（E）^1^HNMR


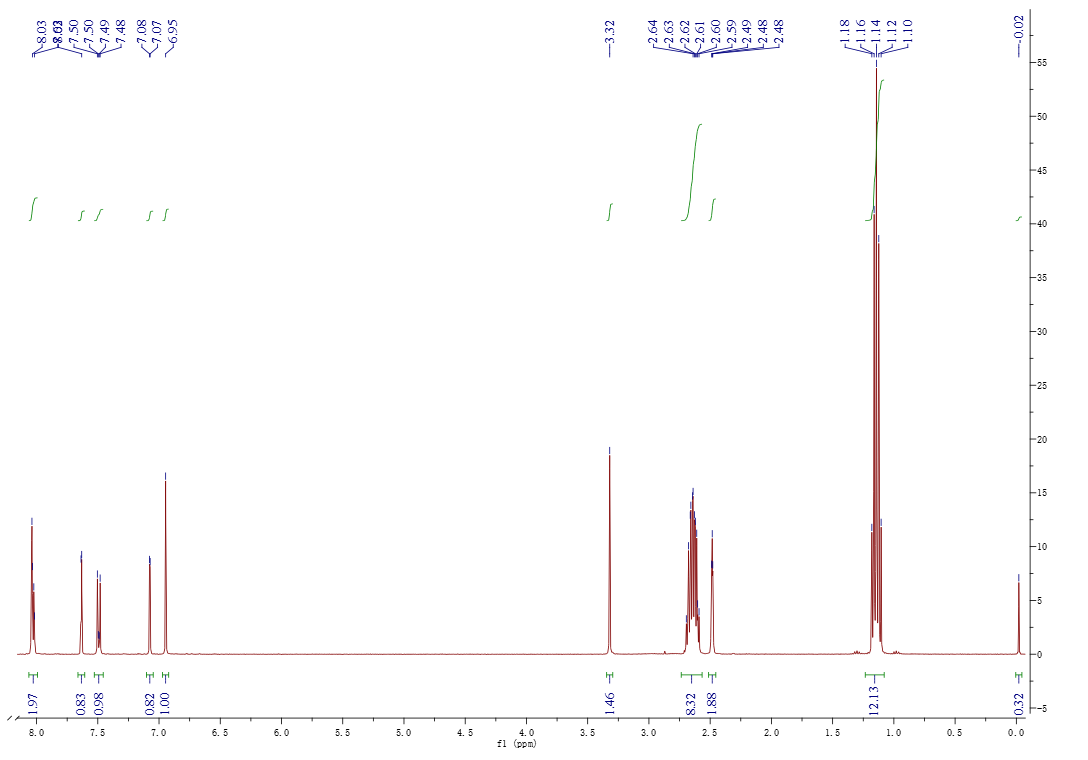


5,7,3 ', 4' - tetra-O-butyrylated luteolin（F）^1^HNMR


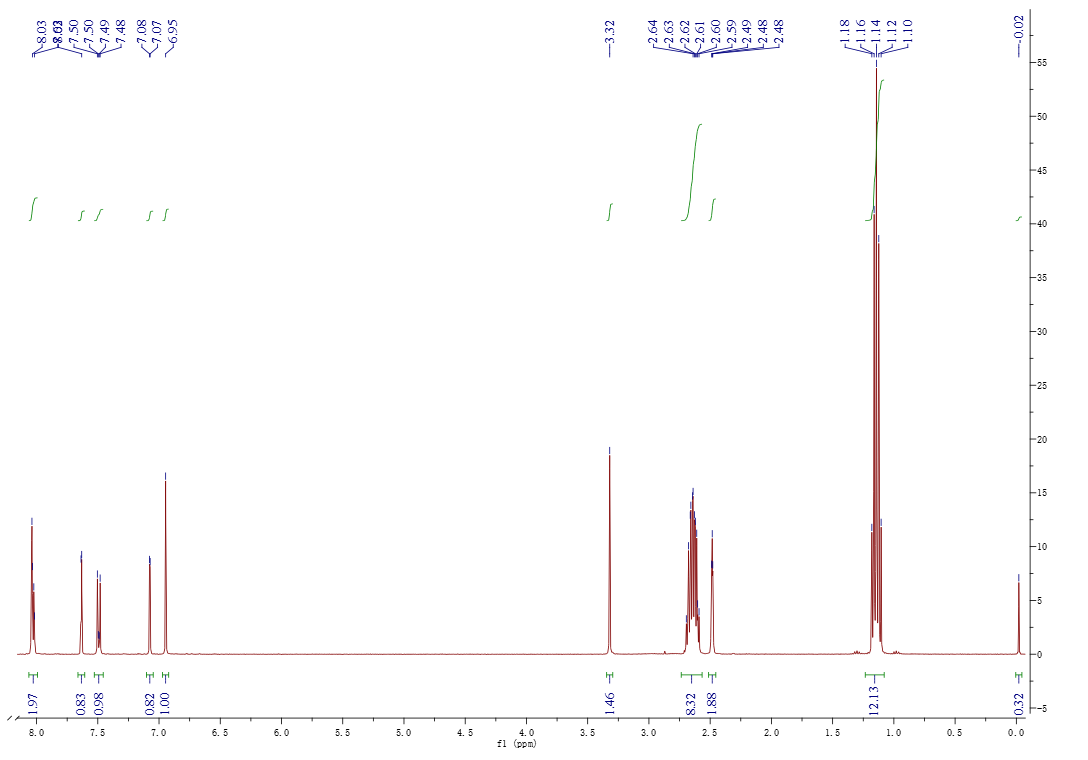

Supplement: Supplementary file 1 [file Data_Sheet_1.docx]
